# A Fuzzy-Set Analysis of Conservative Agriculture Practice Adoption: Role of Farmer Orientations and Attitude

**DOI:** 10.3389/fpsyg.2022.876912

**Published:** 2022-05-30

**Authors:** Naeem Hayat, Abdullah Al Mamun, Anas A. Salameh, Qing Yang, Noor Raihani Zainol, Zafir Khan Mohamed Makhbul

**Affiliations:** ^1^Global Entrepreneurship Research and Innovation Centre, University Malaysia Kelantan, Kota Baharu, Malaysia; ^2^UKM-Graduate School of Business, Univeisti Kebangsaan Malaysia, Bangi, Malaysia; ^3^College of Business Administration, Prince Sattam Bin Abdul Aziz University, Al-Kharj, Saudi Arabia; ^4^Faculty of Entrepreneurship and Business, University Malaysia Kelantan, Kota Baharu, Malaysia

**Keywords:** conservative agriculture practice, orientation, fuzzy sets, innovativeness, configurational theory

## Abstract

Conservative agriculture practice (CAP) adoption literature advocates that adoption is caused by many factors comprising cognitive, social, economic, personal, and CAP-related factors. Evaluating the adoption of CAPs as the outcome is complex and challenging with regression-based models as the systemic interdependencies of the factors offer diverse or varying results. Farmer production and environmental orientations as cognitive stances are notable interpreters of CAP adoption. The appetite level for risk-taking, innovativeness, and trust facilitates the adoption of CAPs. However, a causal-predictive technique should be used to investigate the adoption of CAP. Hence, this study engages in a configuration approach using a fuzzy-set qualitative comparative analysis (fs/QCA) to analyze the patterns of different types of farmers' orientations, personal level of trust on extension services, and innovativeness risk-taking attitude on the intention to adopt CAPs. The analysis is based on the 155-farmer data collected employing a structured interview from Pakistan. The results suggest that a higher level of environment orientation and innovativeness is sufficient to increase the intention to adopt CAPs. Moreover, a higher intention to adopt CAPs is achieved with a lower production orientation, a higher personal level of innovativeness, and a risk-taking attitude of the farmer. The innovativeness can help to develop the intention to adopt the CAPs among the environment and production-oriented farmers. Causal solutions offer a unique understanding that the farmers' environment and production orientation can combine to suggest inclined to adopt CAPs by having an attitude of innovativeness and risk-taking. The causal solutions achieved significant predictive validity in the holdout samples. Policy and farmer-level suggestions were made to raise the intention to adopt CAPs among the farmers.

## Introduction

The global climate shift faced by the world population affects the global industry and agriculture alike (Zhou et al., 2018). With the increasing world population, currently adopted agriculture practices need transference toward sustainable agriculture practices (Wamsler and Brink, 2018). Agriculture contributed about 30% to greenhouse gas (GHG) emissions (Kassam et al., 2018). World agriculture needs to curtail the practices causing GHG emissions and adopt the United Nations Food and Agriculture Organization (UNFAO) recommended conservational agriculture practice (CAPs) to conserve agriculture resources and reduce GHG emissions. It enhances farmer income as well (UNFAO, 2018). CAPs are knowledge-laden practices that require adjustment according to environmental needs with skills and motivation to mitigate the harmful impact of agriculture on world climate (Hayat et al., 2019).

Farmers as agro-entrepreneurs act as decision-makers on their respective farms (Morris et al., 2017). The key determinates of the adoption of CAPs include personal, social, cultural, economic, and institutional factors (Wamsler and Brink, 2018). The development of the intention to adopt CAPs is the first stage toward the likelihood that the adoption of CAPs may occur (Adnan et al., 2017; Mannan et al., 2018). The studies exploring the farmer's intention to adopt the CAPs primarily utilized regression-based models to understand the factors contributing to the farmer's intention to adopt the CAPs (Hayat et al., 2020). However, farmers' personal factors of environment and production orientation are scantly studied (Small et al., 2016; Khatri-Chhetri et al., 2017). The environmental orientation represents the farmer's self-identity with the climate and considers the environment as a fundamental factor of agriculture production. Conversely, the production orientation epitomizes the farmers' self-identity to use commercial farming technology to gain maximum agriculture production. Furthermore, a thorough understanding of the factors influencing CAP adoption intent necessitates acknowledging and approaching the complexities of CAP adoption in allied environmental factors (Bukchin and Kerret, 2018). The configurational approach empowers the researcher to recognize several possible causal solutions to understand intention formation as a more significant phenomenon rather than based on a narrowly drawn set of factors or properties (Farmbach et al., 2016). The configurational approach facilitates the methodological challenges of modeling multiple and complex interrelationships between the factors, producing the configurations as recipes to reach one solution with multiple configurations (Fiss et al., 2013). Multivariate analytical techniques are less adept at capturing the interdependencies among complex systems based on the input and outcome variables. Configurational approaches help theory development by using methods other than the traditional multivariate analysis approaches (Frambach et al., 2015).

This study explores the role of a farmer's environment and production orientation to develop the possible study configurations about the intention to adopt the CAPs. The factors of trust in extension, innovativeness, and risk attitude play a significant role in forming the intention to adopt CAPs. To achieve the configuration challenges of complex adoption choice of the farmers, this study utilizes the fuzzy-set qualitative comparative analysis (fs/QCA), a set based theoretical configurational approach having the ability to handle a higher level of complexity with the multiple causal conditions combined for the outcome to happen (Ragin, 2008). Several recent works recommend the use of QCA and fuzzy-set theory in behavioral and adoption studies and provide new insights into complex causal issues (Salam et al., 2017; Kaya et al., 2020).

In this study, the fs/QCA approach permits to explore the interdependence of the farmers' orientation (environment/production), with trust in extension, innovativeness, and risk-taking rather than only estimating the effect size of the particular orientation and farmer's personal factors on the formation of CAPs adoption intention. The results offer valuable insights into the effect of the farmer's orientation on forming an intention to adopt CAPs and the effect of trust on extension, innovativeness, and risk attitude on the intention to adopt CAPs.

The relevant literature on the CAPs adoption intention is presented in the next section. After the literature exploration, the methodology for this paper was discussed with an explanation of data collection. The analysis for the fsQCA is presented in the later section; the last section of the paper offers the discussion and reports the study's implications and limitations.

## Literature Review

### Conservative Agriculture Practices

CAPs are utilized under various names and classifications. However, in the latter part of the 1990's, these practices were recognized as conservation-oriented and promoted by FAO as an alternative to the intensified agriculture practices (Kassam et al., 2018). CAPs are the farming practices that build on the three core objectives of low soil disturbance, use of soil cover to maintain soil fertility, and crop diversification promoting the farm yield (D'Souza and Mishra, 2018). CAPs are suitable for sustaining farm ecology and mitigating climate change's effect on agriculture production. The imminent climate change and increasing global population stress increase global farm production and mitigate climate change while providing necessary inputs for industry (Zhou et al., 2018). Currently, the CAPs are promoted with economic incentives, which is a short-term solution; in the long run, the farmers' inclination and the right attitude can harness the farmers to adopt the CAPs (Kassam et al., 2018). Mass adoption of CAPs mitigates farm production and reduces the GHGs emissions from agriculture (Hayat et al., 2019). Social and psychological factors play a significant role in forming an intention to adopt the CAPs (Bukchin and Kerret, 2018).

An extensive list of CAPs is available (Hayat et al., 2020). However, few CAPs are regarded as primarily utilized around the world. These practices include no-till, crop rotation, use of bio-fertilizer, direct seeding, and land leveling (UNFAO, 2018). No-till farming is one of the oldest conservative farming practices propagated with the concept of zero disturbance in the farm soil to reduce GHG emissions (D'Souza and Mishra, 2018). No-till enables keeping the necessary farm nutrient particulars on the farm while enabling smooth water movement to enhance farming in natural settings (UNFAO, 2018). Rotating the crops in farming brings soil fertility benefits and brings the necessary microorganism lost due to mono-cropping (Kassam et al., 2018). Crop rotation powers agro-system sustainability and farm productivity (D'Souza and Mishra, 2018). The use of organic fertilizer like animal manure replaced the inorganic fertilizer utilized and is called bio-fertilizer (Kassam et al., 2018). Composting is making fertilizer from animal manure and/or from the other natural sources of nitrogen enriching the farm (Hayat et al., 2020). Direct seeding links with no-till farming practice and brings cost-saving benefits with agronomic and ecological gains (Kassam et al., 2018). Moreover, land leveling enables the farmer to have smooth farmland that enhances the water movement on the farm and brings easiness to the farming operations (Hayat et al., 2020).

### Intention to Adopt CAPs

Intention is the perceived likelihood of an individual taking action or acting in a specific way. In the case of CAP adoption, the intention to adopt is the desire to attempt or adopt the CAPs over time (Adnan et al., 2017). Intention is defined by Hayat et al. (2019) as a proxy for adoption behavior. The willingness and disposition of the farmers to employ CAPs are shown by their intention to embrace them.

### Trust in Extension

Trust is the subjective belief that the other party fulfills their obligations and provides advice to reduce the effects of uncertainty and support in a situation like loss of control (Khandker and Thakurata, 2018). Adoption of CAPs is greatly influenced by the level of trust in the source of information (Fisher et al., 2018). Trust significantly reduces the effects of the associated risk (Hayat et al., 2020). Extension services provide essential information about the CAPs and advice tailored accordingly to the farmer's needs. Farmer trust, in extension, significantly influenced the formation of the intention to adopt CAPs (Zhang et al., 2018).

### Innovativeness

Individuals vary based on their personal inclination toward taking the novel and state-of-art technology in personal and business life (Fisher et al., 2018). A personal predisposition toward the technology instigates the inner acceptance to adopt the innovative technologies and practices (Zhang et al., 2018). A farmer's innovativeness is described as the willingness to change the current farming practices with the new ones based on innate motivation and personal experimentation attitude (Hayat et al., 2020).

### Risk-Taking Attitude

Perception of risk is derived from the outlook of the ambiguity or likely negative outcome of future behavior and is a natural human auspice (Khatri-Chhetri et al., 2017). Adopting new products or technology is seemingly perceived as problematic and inherently considered risky. People with a higher inclination toward risk adopt innovation and new technology earlier than others (Hayat et al., 2020). Moreover, farming is a highly risky profession as farmers usually face weather hardships and natural climates adversely affecting farm productivity. However, among the farmers, the farmer having a higher inclination for risk-taking is more to adopt the CAPs than the risk aversive farmers (Morris et al., 2017).

### Configurational Approach for the Intention to Adopt CAPs Among Farmers

Farmers differ in their attitudes about the environment and productivity as a self-identity, as well as in their agricultural practices preferences (Hayat et al., 2019). Farmers who are concerned about the environment are passionate about it and see it as a crucial aspect in agriculture productivity (Fisher et al., 2018). Climate-conscious farmers are deeply connected to the environment and make every attempt to decrease their agriculture methods' climate-disturbing actions (Small et al., 2016). Farmers' environmental thinking encourages them to employ CAPs and risk lowering farm output as a result of their use (Mannan et al., 2017). Farmers that care about the environment are willing to sacrifice on-farm productivity in order to acquire a personal preference for utilizing CAPs (Zhang et al., 2018).

In contrast to the farmer's environmental orientation, production-oriented farmers seek production-enhancing strategies to achieve higher farm production (Small et al., 2016). Production-oriented farmers are more technology-oriented and have a personal inclination to achieve more production on the farm (Zhang et al., 2018). Farmers with a higher production orientation are concerned more about production and profitability than the conservation of the farm fertility or environment (Small et al., 2016; Morris et al., 2017). In the short term, CAPs may reduce the farm's outcome, and production-oriented farmers are the least inclined toward the CAPs (Partey et al., 2018). However, the production orientation of farmers is also associated with experimenting with new ways of production-enhancing strategies. Therefore, the extension services promote the CAPs and provide enough evidence of the CAPs' impact on farm production (Nakano et al., 2018). The likelihood of the CAPs' adoption among production-oriented farmers remains valid (Small et al., 2016). The production-oriented farmer also has a high inclination for risk-taking (Lal, 2018). Thus, the intention to adopt CAPs among production-oriented farmers may follow the following pattern to develop the intention to adopt CAPs.

Farming is a complex activity and requires multiple inputs at different cropping times (Nakano et al., 2018). Having a good understanding of climatic change is a plus in dealing with the farming challenges (Price and Palis, 2016). Recent times in farming have been marked by the introduction of technology in farming and intensive use of inorganic fertilizer, hybrid seeds, pesticides, and mechanization (Letson, 2017). Farmers who cosseted using the new technology-driven agriculture were known as productive farmers (Small et al., 2016). However, the rise of the climatic issues and their impact on agriculture became an urgent agenda after the issue was taken up by the FAO and the formulation of sustainable development goals (SDGs) (UNFAO, 2018). Adopting CAPs was significantly associated with the farmers' education and environment orientation (Chandra et al., 2018).

**Figure 1:**
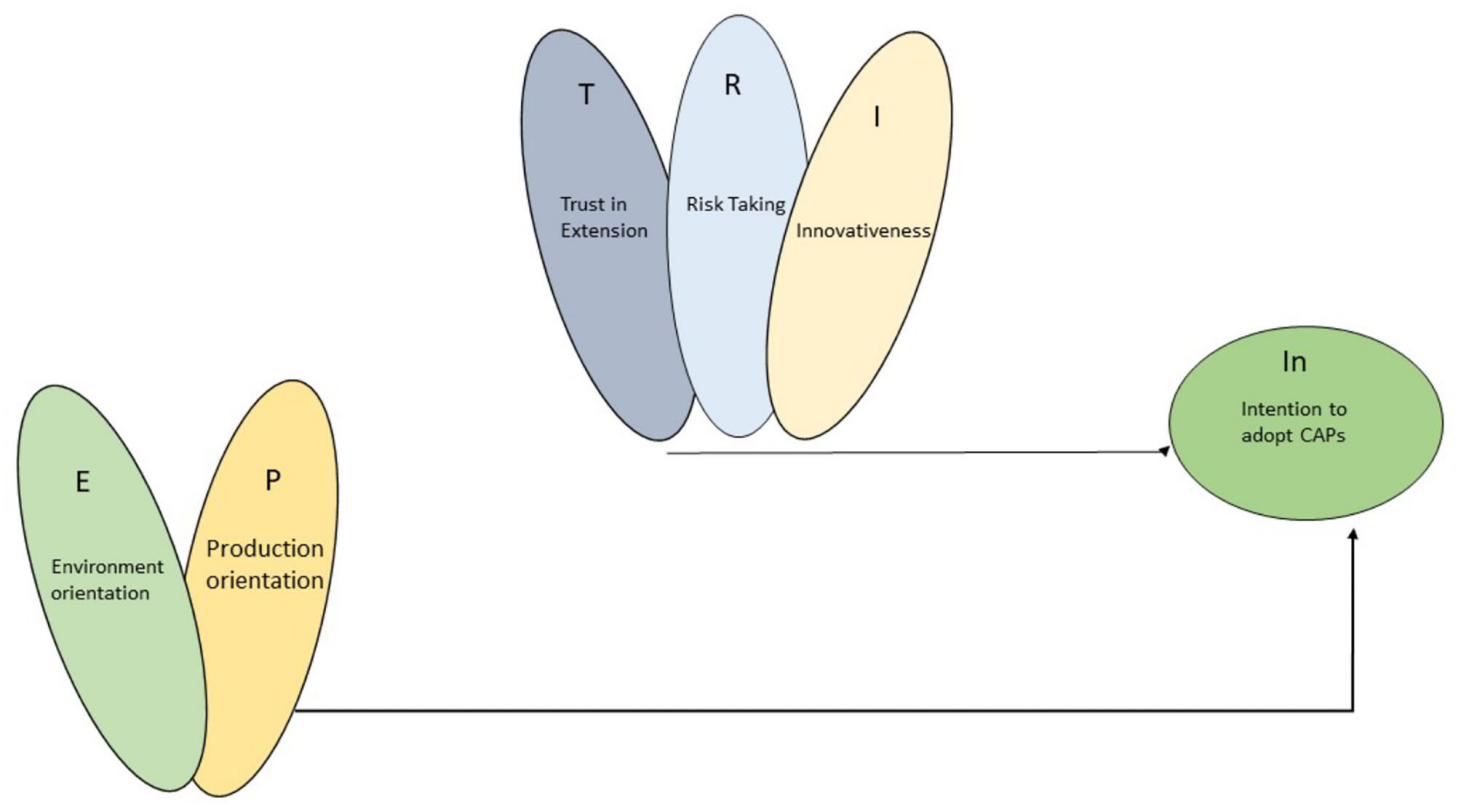
Conceptual framework for complex relationship.

However, significant challenges were posed by the economic objectives of farming (D'Souza and Mishra, 2018). Therefore, it has been established now that the farmers' orientation for the environment impacts the intention to adopt the CAPs (Small et al., 2016). The conceptual framework is offered in Figure 1. However, the impact of the farmer's orientation for environment and production has its influence on the farmer's decision-making in adopting the CAPs.

*Proposition 1: Farmer's environment orientation, combined with the trust in extension, innovativeness, and risk-taking attitude, enables the intention to adopt CAPs*.
*Proposition 2: Farmer's environment orientation and lower trust on extension innovativeness and higher risk-taking attitude allow the intention to adopt CAPs*.
*Proposition 3: Farmer's environment orientation, combined with the lower trust in extension and lower risk-taking attitude with the innovativeness, permits the intention to adopt CAPs*.
*Proposition 4: Farmer's environment orientation, combined with the lower trust in extension, low innovativeness, and lower risk-taking attitude, enables the intention to adopt CAPs*.

However, it is still possible that a farmer with a lack of production orientation and trust in the extension services with an inner inclination for the risk-taking stimulated to try the CAPs. A production orientation may restrict the attempt to try new ways having less reliability than the established farming practices (Small et al., 2016). However, the trust helps to form the intention to try CAPs and take a risk to achieve something for the soil or environmental benefits (Kalungu and Filho, 2018). It may reduce farm production but brings other benefits to the farm (Letson, 2017). Under such conditions, a highly complex mix of factors may emerge and enables the development of the intention to use the CAPs (Khatri-Chhetri et al., 2017). We describe the development of intention to adopt CAPs as follows among production orientation, trust in extension, and risk-taking attitude of the farmers:

*Proposition 5: Farmer's production orientation, combined with the trust in extension, innovativeness, and risk-taking attitude, enables the intention to adopt CAPs*.
*Proposition 6: Farmer's production orientation and lower trust in extension innovativeness, and higher risk-taking attitude allow the intention to adopt CAPs*.
*Proposition 7: Farmer's production orientation, combined with the lower trust in extension and lower risk-taking attitude with the innovativeness, permits the intention to adopt CAPs*.
*Proposition 8: Farmer's production orientation, combined with the lower trust in extension, low innovativeness, and lower risk-taking attitude, enables the intention to adopt CAPs*.

## Method

### Sample and Data Collection

The sample for this study was collected using the convenience sampling method. More than 500 farmers from three villages in central Punjab, Pakistan, were contacted for the study. The villages are situated about 1 h away from the big city of Gujranwala and 2.5 h from the city of Lahore, Pakistan. The study used a structured interview survey to obtain the farmers' opinions and the questions asked about having the rating option. Each interview was completed in no <30 min. The farmers were informed of all the CAPs and the farmers' opinions on eight of the CAPs obtained for the study. The graduate students were appointed to collect data and were trained for this task, as they listened carefully to the farmers (respondents). One hundred and forty-five farmers participated in the field survey interview session, with an overall response rate of 38%. The sample's average age was 54.9 years (a standard deviation of 10.2), and 85% of the sample were male farmers. The average year of farming experience was 21 years (standard deviation = 4.6), and the main crops were wheat, rice, and sugarcane.

### Measurement

The current work utilized the established and verified scales to evaluate the farmers' responses. The data collectors were trained to answer any questions asked by the respondents and explain the questions and answers clearly to the respondents. The follow-up questions were asked from the respondents as well. The question gauged the farmers' decision-making in farming as production orientation: “Do you like to be called a productive farmer as your identity?” The farmer's orientation toward the environment was evaluated by asking, “Do you like to be called an environment-oriented farmer as your identity?” The question items evaluating the production and environmental orientation were taken from Small et al. (2016). Farmer's trust in the extension services was evaluated with five question items, the items borrowed from the work of Dimitriadis and Kyrezis (2010). A sample item was “How do you rate your trust in the extension services provided by the agriculture department?” For the risk-taking attitude, four items were utilized, and the risk-taking questions were taken from Lapple (2013). A sample question item used was, “Are you generally a person who likes to take risks?” The innovativeness was assessed with five question items taken from Agarwal and Prasad (1998). A sample item was “I am interested in using new ways of farming.” The intention to use was gauged by a single question: “I intend to use the CAPs next season.” The item was borrowed from the work of Hayat et al. (2019). The five-point Likert scale was utilized for these question items, where “1” represents does not agree with the statement, and “5” represents fully agree with the statement. The collected data were coded and analyzed using the SPSS 23 software.

### Fuzzy-Set Qualitative Comparative Analysis

The fuzzy-set qualitative comparative analysis (fsQCA) asymmetric modeling technique builds on the combination of fuzzy sets and fuzzy logic (Longest and Vaisey, 2008; Kaya et al., 2020). fsQCA-based modeling handles the complexity as the regression-based modeling based on correlation, and path values cannot fully capture the association between variables (Fiss et al., 2013). fsQCA can identify multiple possible solutions that lead to the same results, which is the opposite of the regression analysis, where only one possible solution leads to one result (Ragin, 2008). A large data set requires multiple regression analysis and structural equation modeling, and high correlation among the variables refers to the collinearity issue and confounding variable not controlled well in the regression-based modeling approaches (Kaya et al., 2020). The variable description is offered in Table 1.

**Table 1:** Set definitions.

**Condition/Outcome**	**Variable**	**Set membership**
Outcome	Intention to adopt CAP	Farmers with higher intention to adopt CAPs.
Antecedent condition	Environment orientation	Farmer with environment orientation.
Antecedent condition	Production orientation	Farmer with production orientation.
Antecedent condition	Trust on extension	A farmer has trust in the extension.
Antecedent condition	Risk-taking attitude	A farmer has a risk-taking attitude.
Antecedent condition	Innovativeness	A farmer is innovative in updating new farming technologies.

Furthermore, the fsQCA approach can handle the positive and negative logic, as the reliance on one-sided logic is misleading. Taking all the possible combinations that can lead to a positive outcome may not lead to a negative outcome, confirming the causation (Fiss et al., 2013). The fsQCA facilitates identifying sufficient and necessary conditions using Boolean algebra from the set of observations (Longest and Vaisey, 2008; Ragin, 2008).

Four stage analysis process is postulated to use in the fsQCA. First, the original study variables were five-point Likert scales rescaled from 0 to 1, where 0 denotes the full non-membership of the set and 1 shows the complete set membership. The process is named calibration (Fiss et al., 2013). We checked the skewness of our uncalibrated data; the data show the skewness was 1, and the kurtosis was <2. It depicts the normal distribution of the data (Kaya et al., 2020). The second step requires the necessity analysis or labeled as configurational elements. A condition is termed as necessary when its consistency score is above 0.90 (Ragin, 2008). Necessity analysis denotes the proportion of fuzzy-set scores in all the cases that are less than or equal to the corresponding scores in the study outcome (Fiss et al., 2013).

The third step is fsQCA analysis to achieve the trust table algorithm; the trust table produces the 2^∧^k rows, where k shows the number of outcomes utilized in the analysis, and row shows every possible combination among the causal settings of the study (Ragin, 2008). For the sample over 150, the outcome score of 3 was suggested as acceptable (Longest and Vaisey, 2008; Fiss et al., 2013). Here, consistency refers to the degree to which cases match the set-theoretic relationship expressed in a solution, and the threshold consistency is set at 0.75 (Ragin, 2008).


$$\begin{array}{l} \text{Consistency}\left(\text{CO}{\text{N}}_{\text{i}}\right)=\left[\text{min}\left(\text{CO}{\text{N}}_{\text{i}},{\text{Y}}_{\text{i}}\right)\right]/\left(\text{CO}{\text{N}}_{\text{i}}\right) \end{array}$$

In the fourth stage, the final configuration analysis enables identifying the best antecedent conditions that lead to the higher achievement of the outcome (Fiss et al., 2013). fsQCA analysis offers three solutions, that is, complex, parsimonious, and intermediate (Salam et al., 2017). The complex solution offers the all-possible configurations or combinations of input variables that lead to an outcome (Longest and Vaisey, 2008; Ragin, 2008). This type of solution is needlessly complex and impractical as the causal configuration is not possible. The intermediate solution offers a mix of vital configurations that are parsimonious and complex (Kaya et al., 2020). The parsimonious solution offers only the vital configurations that lead to either easy or difficult outcomes (Fiss et al., 2013). The antecedent conditions with consistency at or above 0.90 are necessary to achieve a higher outcome (Ragin, 2008). The core conditions depict the causal link of input that leads to the outcome. The coverage describes the % of the explanation that offers the causal combination in the final solution (Ragin, 2008).

**Figure 2:**
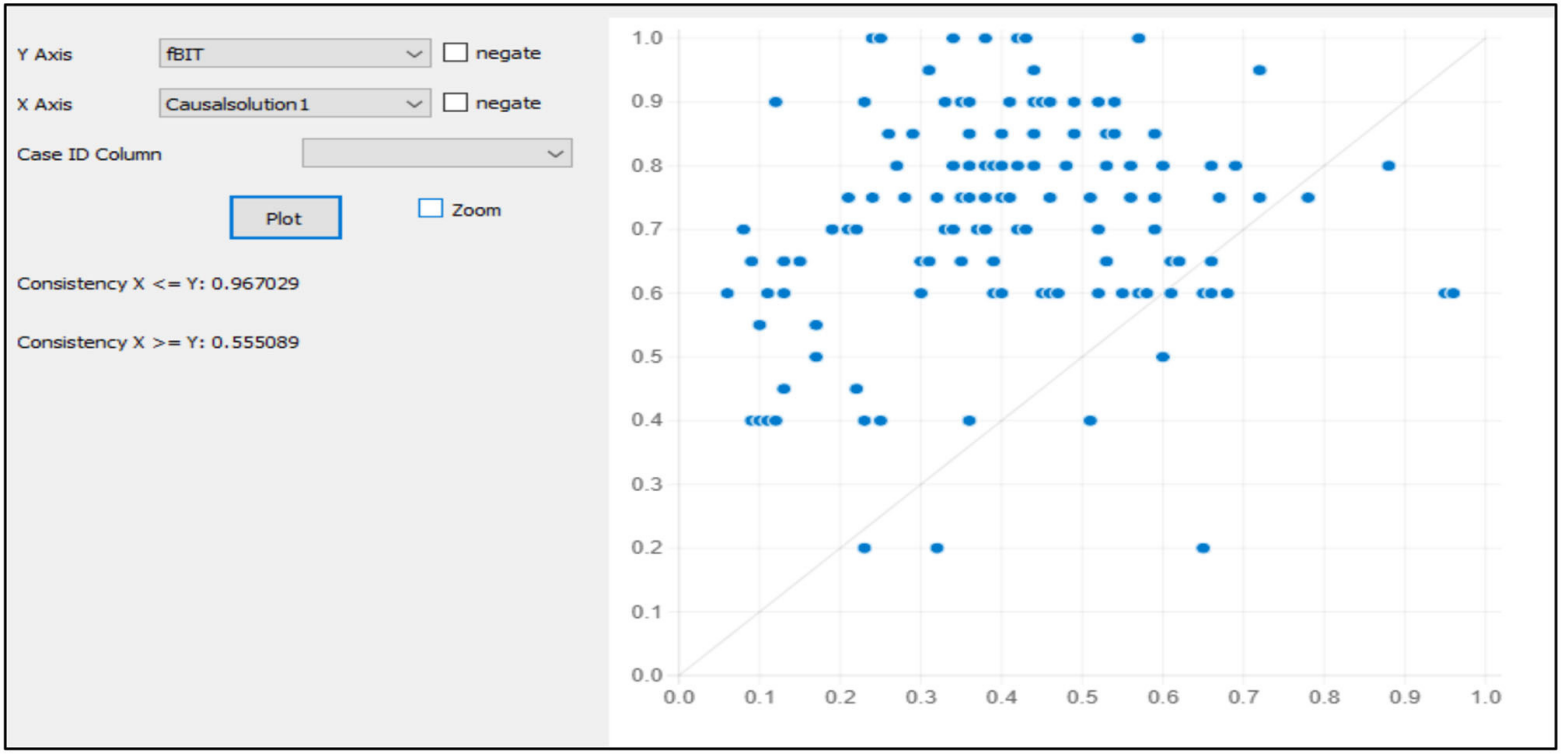
Fuzzy plot for model 1 within holdout sample.

**Figure 3:**
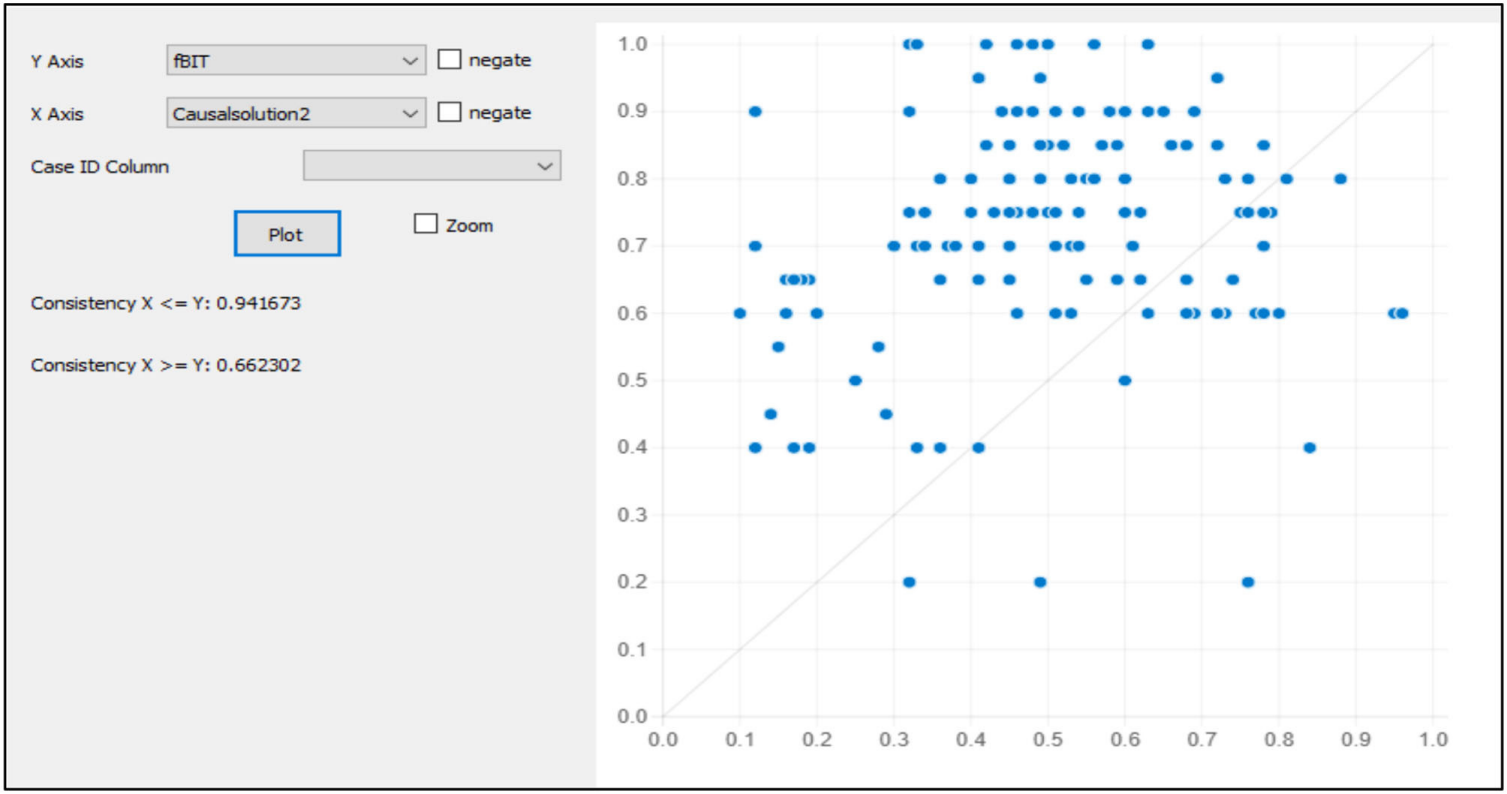
Fuzzy plot for model 2 with holdout sample.

Testing for the causal solution's predictive validity is vital to depict the goodness of the causal solutions for predicting the dependent variable with the holdout sample (Pappas and Woodside, 2021). To evaluate the predictive validity of the causal model, first of all, the data must be split randomly into the primary sample data and holdout sample data. The causal solution was obtained by running the fsQCA created as a variable in the holdout sample. The computation of the causal solution in the holdout sample is then plotted against the outcome variable (see Figures 2, 3). The high consistency score of 0.80 and coverage depict the validity of the casual solutions.

## Results

The first step in the data preparation for fuzzy-set analysis was calibration (Kaya et al., 2020). Collected data measurement transformed into sets is easy and performed by the direct method prescribed by Ragin (2008). A fuzzy-set measurement assesses the membership in the set of the respective variables after calibration. The study variables, i.e., environment orientation, production orientation, trust in extension, innovativeness, risk-taking attitude, and intention to adopt CAPs, the five-value fuzzy-set values for full non-membership, the crossover point, and full membership are 0.05, 0.50, and 0.95, respectively, for all the variables adopted. All analyses were performed with Stata 16 software package, utilizing the fuzzy commends (Longest and Vaisey, 2008). Table 2 offers the mean and standard deviation with the calibration of the study variables. In Table 3, the correlation and the construct level of Cronbach's alpha value are provided.

**Table 2:** Measures properties and calibration.

**S. No**.	**Variable**	**Range**	**Mean**	**S.D**.	**Calibration**
1.	Environment orientation	1–5	3.19	0.97	0.05, 0.35, 0.50, 0.75, 0.95
2.	Production orientation	1–5	3.22	0.71	0.05, 0.35, 0.50, 0.75, 0.95
3.	Trust on extension	1–5	3.05	0.68	0.05, 0.35, 0.50, 0.75, 0.95
4.	Risk taking	1–5	3.89	0.91	0.05, 0.35, 0.50, 0.75, 0.95
5.	Innovativeness	1–5	3.35	0.97	0.05, 0.35, 0.50, 0.75, 0.95
6.	Intention to adopt CAP	1–5	3.56	0.82	0.05, 0.35, 0.50, 0.75, 0.95

*Author calculations*.

**Table 3:** Correlation matrix.

	**Construct**	**No. of items**	**Cronbach's Alpha**	**1**	**2**	**3**	**4**	**5**	**6**
1.	Environment orientation	1	1	1					
2.	Production orientation	1	1	−0.12	1				
3.	Trust on extension	5	0.785	0.14	0.28	1			
4.	Risk taking attitude	4	0.700	0.29	0.35	0.24	1		
5.	Innovativeness	5	0.863	0.65	0.57	0.46	0.59	1	
6.	Intention to adopt CAPs	1	1	0.19	0.22	0.33	0.47	0.49	1

*Author calculations*.

After calibrating the data values into sets, a test for the necessary condition needs to perform to confirm the consistency threshold of 0.90 as a necessary condition for each set to establish that no condition passed the threshold (see Table 4). A truth table with 2^k^ rows is the second set to perform to organize the configuration of conditions. “K” represents the causal condition opted for analysis. For our study, the number of conditions is “4” and 2^4^ = 32. The minimum number of cases is three, and the minimum acceptable row consistency is set at 0.80 (Longest and Vaisey, 2008; Ragin, 2008).

**Table 4:** Analysis of necessary conditions for higher intention to use CAPs.

	**High intention**		**Low intention**	
	**Consistency**	**Coverage**	**Consistency**	**Coverage**
Production orientation	0.945	0.412	0.674	0.818
Environment orientation	0.935	0.414	0.706	0.822
Trust in extension	0.945	0.417	0.681	0.798
Risk taking	0.961	0.426	0.703	0.812
Innovativeness	0.945	0.430	0.686	0.744

*Author calculations*.

Moreover, increasing the PRI (proportional reduction in consistency) values decreases the model coverage without improving the consistency scores (Fiss et al., 2013). Table 4 depicts the analysis of necessary conditions. The results show that the no casual condition achieves the necessary condition status as the consistency for presence and absence of the casual conditions consistency values ranges between 0.559 and 0.836. Therefore, a causal combination of conditions can lead to a higher intention to adopt the CAPs. The consistency scores for input variables leading to higher and lower CAPs adoption intention are depicted in Table 4.

Fuzzy-set analysis results are presented as a postulated method proposed by Ragin (2008). The analysis reported in Table 5 shows that a capital (X) specifies core conditions, and a small (x) represents the condition's absence. Blank space indicates an extreme condition that may not affect the outcome, that is, the intention to adopt CAPs in our case. Coverage scores indicate the importance of the configuration as to how many study cases fit this path to the outcome, that is, the intention to adopt CAPs (Kaya et al., 2020). The solution coverage acts like R^2^ in traditional regression, explaining the % of the outcome with input variables (Ragin, 2008). The consistency threshold is recommended at 0.80.

**Table 5:** Configuration for study propositions.

**Propositions**	**1**	**2**	**3**	**4**	**5**	**6**	**7**	**8**
**Orientation**								
Environment orientation (E)	***X***	***X***	***X***	***X***	*	*	*	*
Production orientation (P)	*	*	*	*	***X***	***X***	***X***	***X***
**Attitude**								
Trust on extension (T)	***X***	x	x	x	**X**	x	x	X
Risk-taking (R)	***X***	***X***	x	x	**X**	***X***	x	X
Innovativeness (I)	***X***	***X***	**X**	x	**X**	***X***	***X***	X
Consistency	0.914	0.902	0.895	0.854	0.875	0.814	0.806	0.707
Raw coverage	0.306	0.282	0.284	0.294	0.254	0.260	0.258	0.266
Unique coverage	0.306	0.282	0.284	0.294	0.254	0.260	0.258	0.266

(X) represents the presence of the factors, (x) shows the low level of the factors, and (*) shows the absence of the factor.

For the proposed configuration, configuration 1 combines the presence of a farmer's environment orientation with the trust in extension, innovativeness, and risk-taking attitude. This combination describes the absence of production orientation. Environment orientation and the absence of production orientation is the core condition for this configuration. The proposed configuration shows high consistency and coverage (consistency = 0.914, coverage = 0.306).

The second proposed configuration combines the farmers' environment orientation with risk-taking and innovativeness but the absence of trust in extension. The proposition 2 shows acceptable consistency and coverage (consistency = 0.902, coverage = 0.282). The third proposed configuration combines the farmers' environment orientation with innovativeness, lack of trust in extension, and a risk-taking attitude. This proposed formation shows high consistency and coverage (consistency = 0.895, coverage = 0.284). The fourth proposed configuration combines the farmers' environment orientation with the absence of trust in extension, risk-taking, and innovativeness. The proposition 4 shows acceptable consistency and coverage (consistency = 0.854, coverage = 0.294).

Furthermore, proposed configuration 5 combines the presence of a farmers' production orientation with the trust in extension, innovativeness, and risk-taking attitude. The proposed configuration 5 shows high consistency and coverage (consistency = 0.875, coverage = 0.254). The sixth proposed configuration combines the farmers' production orientation with risk-taking and innovativeness but the absence of trust in extension. The proposition 6 shows acceptable consistency and coverage (consistency = 0.814, coverage = 0.260). The seventh proposed configuration combines the farmers' production orientation with innovativeness, absence of trust in extension, and a risk-taking attitude. This proposed formation shows acceptable consistency and coverage (consistency = 0.806, coverage = 0.258). The eighth proposed configuration combines the farmers' production orientation with the absence of trust in extension, risk-taking, and innovativeness. The proposition 8 shows unacceptable consistency (consistency = 0.707, coverage = 0.266).

### Other Possible Causal Configuration

Conducting additional analysis in QCA helps to identify configurations that might lead to the presence of high performance, i.e., the intention to adopt CAPs in our case (Ragin, 2008). Causal asymmetry is achieved through these additional analyses and the unique advantage of the QCA method (Salam et al., 2017). As a configuration that leads to an outcome that may be different from conditions as proposed in any study, the current research work attempts to examine the high intention to adopt CAPs. The three intermediate solutions offered by the fsQCA analysis can lead to a higher intention to use the CAPs (Fiss et al., 2013). The first causal solution 1 shows the presence of higher environment orientation and profit orientation, with risk-taking and innovativeness attitude, is necessary to build the positive intention to adopt the CAPs and that the 37.8% of the intention to adopt CAPs can be explained by causal solution 1 (Salam et al., 2017).

The causal solution 2 shows higher environment orientation, risk-taking, and innovativeness to build the intention to adopt the CAPs. The 35.6% of the intention to adopt CAPs can explain causal solution 2 (Ragin, 2008).

In causal solution 3, personal innovativeness is necessary to instigate the intention to use the CAPs. The solution can explain the 28.8% of the intention to adopt CAPs in the study sample. However, the consistency for solution 3 is less than the 0.75 cutoffs (Kaya et al., 2020). The overall solution consistency for the causal solutions is more than the consistency threshold. The solution coverage of the three possible causal solutions can explain 56.1% of the outcome, i.e., the intention to adopt CAPs. The configuration results are offered in Table 6.

**Table 6:** Configuration for study propositions.

**Causal solution**	**1**	**2**	**3**
**Orientation**			
Environment orientation (E)	***X***	***X***	*
Production orientation (P)	***X***	*	*
**Attitude**			
Trust on extension (T)	*	*	*
Risk-taking (R)	***X***	***X***	*
Innovativeness (I)	***X***	***X***	***X***
			
Consistency	0.925	0.909	0.601
Raw coverage	0.378	0.356	0.288
Unique coverage	0.102	0.083	0.094
**Overall solution consistency**	0.760		
**Overall solution coverage**	0.561		

(X) represents the presence of the factors, (x) shows the low level of the factors, and (*) shows the absence of the factor.

### Testing for Predictive Validity

Testing the casual solutions' predictive validity is vital. Checking the casual solutions' predictive validity depicts how well the causal solution predicts the dependent outcome (Salam et al., 2017). Evaluating the predictive validity shows the model's goodness for prediction reasons (Pappas and Woodside, 2021). The causal solutions 1 and 2 were tested with the holdout sample.

We first compute the configuration depicted in causal solution 1 with the holdout sample. Causal solution 1 shows that environment orientation, production orientation, risk-taking, and innovativeness promote the intention to adopt the CAPs. The new variable is computed as causal solution 1 and then plotted against the outcome variable, that is, the intention to adopt the CAPs (Pappas and Woodside, 2021). The results of the fsQCA plot show the high consistency of the causal solution 1 (consistency = 0.967 or 96%) and have coverage of (0.55, 55%) with the causal solution 1 for the intention to adopt CAPs.

Then, we compute the configuration as depicted in causal solution 2 with the holdout sample. Causal solution 2 shows that environment orientation, risk-taking, and innovativeness can promote the intention to adopt the CAPs. The new variable is computed as causal solution 2 and then plotted against the outcome variable, i.e., the intention to adopt the CAPs (Pappas and Woodside, 2021). The results of the fsQCA plot show the high consistency of causal solution 2 (0.944 or 94%) and have the coverage of 0.660 or 66% with causal solution 2 for intention to adopt CAPs.

## Discussion and Conclusion

### Intention to Adopt CAPs With Different Farmers' Orientations and Attitudes

The current work highlights the adoption of CAPs as a complex function based on the farmer's orientation (environment and production) and the trust in extension, innovativeness, and risk-taking as attitudes facilitating the intention to adopt CAPs among the farmers in Pakistan. The study forwarded four propositions that farmers' environmental orientation offers sufficient explanation to build the intention to adopt CAPs. Farmers' trust in extension, risk-taking, and innovation builds the sufficient intention to adopt CAPs when farmers trust their extension. The first four propositions can explain 28.2–30.6% of the farmers' intention to adopt the CAPs.

A farmer's production orientation and a farmer's attitude forming the intention to adopt CAPs are estimated with the proposition 5 to 8. The farmers' production orientation sufficiently explains the intention to adopt the CAPs when farmers trust extension, risk-taking, and innovativeness. However, the farmers' production orientation offers fewer explications of the intention to adopt CAPs than the environmental orientation. The explaining power ranges from 26.6 to 25.4%.

The study's findings support the notion that farmers with an environmental orientation are more likely to implement CAPs than farmers with a production orientation. Nevertheless, our study confirms with Chandra et al. (2018) that the farmers' environmental orientation significantly explains the farmers' intention to adopt the CAPs. However, most of the literature postulates that the farmers' profit orientation leads to the non-adoption of the CAPs. Farmers with a profit orientation think CAPs are costly and not fit to earn a good profit (Morris et al., 2017).

We take advantage of the fsQCA and look for our study's most parsimonious configuration solution. The first causal solution describes how farmers with environmental and production orientations, as well as a risk-taking and innovative attitude, are more likely to adopt CAPs when trust in extension is lacking. Our study matches the findings posted by Hayat et al. (2020) that farmers with a risk-taking mindset are more inclined to use the CAPs. Furthermore, Zhang et al. (2018) postulated that the farmers' innovativeness helps harness the intention to use the CAPs. The current causal solution suggests that farmers with production orientation and environmental orientation take an interest in using the CAPs with the appropriate level of risk-taking and innovativeness.

The second causal solution offers environmental orientation with risk-taking and innovativeness, promoting the intention of adopting the CAPs. The causal solution coincides with the findings posted by Small et al. (2016) that the farmer's environmental orientation plays a prime role in adopting CAPs. Farmers' risk-taking and innovativeness solely influence their intention to implement CAPs, which is consistent with the findings presented by Morris et al. (2017) and Hayat et al. (2020), respectively. The casual solution shows that farmers with environmental orientation, risk-taking attitude, and innovativeness show more inclination toward adopting the CAPs.

The third causal solution demonstrates that the farmers' innovativeness sufficiently predicts the intention to adopt CAPs. The result supports the argument that farmers still tend to adopt the CAPs without having the production or environmental orientation and that farmers' innovativeness is enough to make them positively inclined to adopt the CAPs.

These three causal solutions can explain 56.1% of the intention to adopt CAPs and acceptable solution consistency. The fsQCA offers a unique causal solution in that farmers' environment and production orientation can inculcate the intention more strongly than the single orientation. The causal solution (1) exceeds in explaining the power of the proposed proposition of the study. The out-of-sample predictive validity of casual solutions 1 and 2 supports using the casual solution in out-of-sample data. The results offer validity to the causal solutions 1 and 2.

### Implications

This study contributes to the CAPs adoption literature and practices. This study's findings suggest that a farmer's environment and production orientation meaningfully help to construct the intention to adopt CAPs. The current research work offers sufficient support to believe in working on building farmer environment orientation as it may help develop intention for adopting CAPs.

This study contributes to the CAPs adoption literature in two ways. First, farmer orientation for environment and orientation for production are not opposed to each other but coexist in a way to develop the intention to adopt CAPs. Progressive farmers can be environmentally oriented and able to achieve profit simultaneously. However, the progressive farmers are different from the average farmers that only concentrate on profit or the environment. Policies require to uplift the number of progressive farmers and offer them educational and financial support to achieve agriculture sustainability. Improving the farmers' production orientation and building environmental awareness can help to promote the adoption of CAPs. Risk-taking and innovativeness as attitudes play a vital role in the higher intention to adopt the CAPs. Promoting a positive attitude is necessary to instigate the positive intention to use the CAPs as a farming strategy that can help the farmers achieve higher farm productivity by reducing the GHGs from agriculture.

Second, the farmers' trust in extension, innovativeness, and personal risk-taking attitude plays a substantial role in developing the intention to adopt CAPs but to fluctuating degrees. The study findings presented an interactive role of innovativeness, trust in extension, and risk-taking in developing the intention to adopt CAPs. Therefore, this study points toward the importance of farmer orientation and the complex nature of farmers' attitudes interacting to build the intention to adopt the CAPs. The current study results postulate that the alternative farmer orientations (i.e., environment and production) affect the formation of an intention to adopt the CAPs with the combined effect of the farmer's level of trust and appetite for risk-taking. Moreover, the complex relationships depict the ambiguity of the previous research findings based on the contexts. More broadly, the complex pattern of relationships is more subtle and associated with unique ambiguity to explore and capture the interrelationship of the phenomenon. Therefore, it is vital to utilize the fsQCA, a methodological toolkit with correlation data at hand with the set-theoretical and configuration approach.

### Limitations and Future Research Opportunities

Adoption decisions are complex, and individual personal orientations and attitudes play a significant role in forming an inclination to adopt innovative and novel technologies and practices. Traditional analysis techniques are insufficient to address the complexity and multiple causal conditions that lead to the optimal solution for a causal combination of factors. fsQCA offers a unique analysis technique to sufficiently address the complexity and offers the causal combination that offers multiple causal solutions leading to a higher optimal outcome.

The current study is associated with three pertinent, significant limitations. First, the study was cross-sectional and single-sourced, with limited respondents. We suggest that the future study may utilize the longitudinal survey design to estimate the different configurations by which farmers develop the intention to adopt the CAPs. Furthermore, the current study used the intention to measure the CAPs' adoption rather than their actual adoption. Furthermore, experience with CAPs can shape the various aspects of attitude that can lead to the intention and adoption of CAPs. Future studies need to explore the effect of CAPs' experience on the intention formation and adoption of CAPs among farmers from emerging and developed economies.

## Data Availability Statement

The raw data supporting the conclusions of this article will be made available by the authors, without undue reservation.

## Ethics Statement

Ethical review and approval was not required for the study on human participants in accordance with the local legislation and institutional requirements. The patients/participants provided their written informed consent to participate in this study.

## Author Contributions

AS, QY, NZ, and ZM: conceptualisation, designing the instrument, and writing–original draft. NH and AA: data collection, formal analysis, and writing–revision and amendment. All authors contributed to the article and approved the submitted version.

## Conflict of Interest

The authors declare that the research was conducted in the absence of any commercial or financial relationships that could be construed as a potential conflict of interest.

## Publisher's Note

All claims expressed in this article are solely those of the authors and do not necessarily represent those of their affiliated organizations, or those of the publisher, the editors and the reviewers. Any product that may be evaluated in this article, or claim that may be made by its manufacturer, is not guaranteed or endorsed by the publisher.
